# Survey of causative agents for acute respiratory infections among patients in Khartoum- State, Sudan, 2010–2011

**DOI:** 10.1186/1743-422X-10-312

**Published:** 2013-10-25

**Authors:** Khalid A Enan, Takeshi Nabeshima, Toru Kubo, Corazon C Buerano, Abdel Rahim M El Hussein, Isam M Elkhidir, Eltahir AG Khalil, Kouichi Morita

**Affiliations:** 1 Central Laboratory, Ministry of Science and Technology, P.O. Box 7099, Khartoum, Sudan; 2 Department of Virology, Institute of Tropical Medicine, Nagasaki University, Nagasaki 852-8523, Japan; 3 Department of Molecular Epidemiology, Institute of Tropical Medicine, Nagasaki University, Nagasaki 852-8523, Japan; 4 Animal Resources Research Corporation, P.O. Box 610, Khartoum, Sudan; 5 Department of Microbiology and Parasitology, Faculty of Medicine, University of Khartoum, P.O. Box 8067, Khartoum, Sudan; 6 Institute of Endemic Diseases, University of Khartoum, P.O. Box 45235, Khartoum, Sudan

**Keywords:** Influenza viruses, Human respiratory syncytial virus, Rhinovirus, Adenovirus, Epidemiology, Sudan

## Abstract

**Background:**

This study was carried out to determine causative agents of acute respiratory illness of patients in Khartoum State, Sudan.

**Methods:**

Four hundred patients experiencing respiratory infections within January-March 2010 and January-March 2011 were admitted at Khartoum Hospital and had their throat swab samples subjected to multiplex real-time RT-PCR to detect influenza viruses (including subtypes) and other viral agents. Isolation, nucleotide sequence and phylogenetic analysis on some influenza viruses based on the HA gene were done.

**Results:**

Out of 400 patients, 66 were found to have influenza viruses (35, 27, 2, and 2 with types A, B, C, and A and B co-infections, respectively). Influenza viruses were detected in 28, 33 and 5 patients in the age groups <1, 1–10, and 11–30 years old, respectively but none in the 31–50 years old group. Out of 334 patients negative for influenza viruses, 27, 14, and 2 were positive for human respiratory syncytial virus, rhinovirus and adenovirus, respectively. Phylogenetic tree on influenza A (H1N1) pdm09 subtype shows that Sudan strains belong to the same clade and are related to those strains from several countries such as USA, Japan, Italy, United Kingdom, Germany, Russia, Greece, Denmark, Taiwan, Turkey and Kenya. Seasonal A H3 subtypes have close similarity to strains from Singapore, Brazil, Canada, Denmark, USA and Nicaragua. For influenza B, Sudan strains belong to two different clades, and just like influenza A (H1N1) pdm09 and A H3 subtypes, seem to be part of worldwide endemic population (Kenya, USA, Brazil, Russia, Taiwan and Singapore).

**Conclusions:**

In Sudan, the existence of respiratory viruses in patients with acute respiratory infection was confirmed and characterized for the first time by using molecular techniques.

## Background

Influenza and other respiratory tract infecting viruses are a common cause of upper and lower respiratory tract infection and are responsible for morbidity and mortality especially in the elderly and in high-risk groups such as immunocompromised patients and those with chronic diseases [1,2]. Influenza viruses have been well studied in developed countries. However, the epidemiology of influenza and the hospitalization rates associated with the illness may be different in sub-Saharan Africa for several reasons [3,4].

Meager information is available on the epidemiology of influenza in Sudan. This is mainly due to lack of laboratory facilities and expertise. Influenza is also perceived as a mild disease in a country like Sudan where other causes of fever such as malaria, kala azar, and typhoid are highly prevalent [5]. The first isolation of influenza virus in Sudan was carried out by Salim in 1970, who reported on the isolation of haemagglutinating agents from throat gargles by using chicken embryos [5].

Although, surveillance data from the African continent has increased substantially in the past five years, they are inadequate to allow for a thorough understanding of influenza virus circulation patterns on the continent and their associated morbidity and mortality, or to provide information for influenza control strategies [6]. In the Afriflu meeting that was held in June 2010 in Marrakesh, Morocco, influenza specialists and public health experts pledged to follow concrete measures to bridge the knowledge gap on the burden of influenza in Africa [6]. One of the key measures recommended was the reinforcement of routine influenza surveillance capacity both from an epidemiological and a virological standpoints.

The present study aimed to identify human influenza viruses circulating in Sudan from January to March 2010 and January to March 2011, periods at which high incidence of acute respiratory infections are usually recorded [5]. This was accomplished by subjecting throat swab samples taken from suspected influenza patients to multiplex real-time RT-PCR and subjecting some identified influenza viruses for nucleotide sequence. Samples negative for influenza viruses were then tested for other common respiratory tract-infecting viruses namely human respiratory syncytial virus (RSV), human metapneumovirus (hMPV), rhinovirus, and adenovirus.

## Results

### Influenza viruses and other respiratory viruses

During the study period, 400 patients were enrolled. Three hundred and sixty eight 368 (92%) were children between the ages of <1 and 10 years. The median length of hospitalization was 2 days. Influenza viruses were detected in 66 (16.5%) patients (Table 1). Out of 66 patients, 35 (53.0%) were detected with influenza type A virus, 27(40.9%) with influenza type B, 2 (3%) with influenza type C and 2 (3%) with a co-infection of influenza types A and B. Out of the 37 patients positive for influenza A due to single or co-infection, 28 (75.7%) were infected with A(H1N1)pdm09, 8 (21.6%) with H3 subtypes and 1 (2.7%) unsubtypeable. While both subtypes were detected from patients during 2011 collections, no subtype H3 was detected in 2010 collections (Table 2).

**Table 1:** Total number of patients by age and the distribution of influenza viruses in 66 patients

		**Number of patients with influenza viruses (%)**					
Age group in years	Number of patient (%) [%]^1^	A	B	C	AB	Total negative (%)	Total positive (%) [%]^2^
<1	164 (100) [41.0]	19 (54.3)	8 (29.6)	1 (50.0)	0 (0)	136 (82.9)	28 (17.1) [42.4]
1-10	204 (100) [51.0]	13 (37.1)	17 (63.0)	1 (50.0)	2 (100)	171 (83.8)	33 (16.2) [50.0]
11-30	27 (100) [6.8]	3 (8.6)	2 (7.4)	0 (0)	0 (0)	22 (81.5)	5 (18.5) [7.6]
31-50	5 (100) [1.2]	0 (0)	0 (0)	0 (0)	0 (0)	5 (100)	0 (0)
Total	400 (100) [100]	35 (8.8) {53.0}^3^	27 (6.8) {40.9}	2 (0.5) {3.0}	2 (0.5) {3.0}	334 (83.5)	66 (16.5) [100] {100}

^1^Percent inside bracket found on the second column is % out of 400 patients.

^2^Percent inside bracket found on the last column is % out of 66 patients.

^3^Percent inside curly bracket found on the last row is % out of 66 patients.

**Table 2:** Number of patients with influenza viruses detected in 2010 and 2011

**Year**	**Number of patients with influenza viruses**				**Total number of**	**Total number of**	**Number of patients with specific**		
					**patients with**	**patients with**	**subtype of influenza A**		
	A	B	C	AB	Influenza A,B,C	Influenza A	A (H1N1) pdm09	H3	Unsubtypeable
2010	4	8	0	0	12	4	4	0	0
2011	31	19	2	2	54	33	24	8	1
Total	35	27	2	2	66	37	28	8	1

Out of 334 patients that were negative for influenza viruses, 43 (13%) were found positive for other respiratory viruses. Out of these positives, 27(62.8%), 14 (32.6%), and 2 (4.6%) had RSV, rhinovirus and adenovirus infections, respectively (Table 3). No patient was found positive for hMPV.

**Table 3:** Patients grouped by age and detected with other respiratory viruses

**Age group in years**		**Number of patients (%)**			
	**RSV**	**hMPV**	**Rhinovirus**	**Adenovirus**	**Total**
<1	17 (81.0)	0 (0)	4 (19.0)	0 (0)	21 (48.9)
1-10	9 (45.0)	0 (0)	9 (45.0)	2 (10.0)	20 (46.5)
11-30	1 (50.0)	0 (0)	1 (50.0)	0 (0)	2 (4.6)
31-50	0 (0)	0 (0)	0 (0)	0 (0)	0 (0)
Total	27 (62.8)	0 (0)	14 (32.6)	2 (4.6)	43 (100)

Based on age group, the distribution of 66 patients positive for influenza viruses were 28 (42.4%), 33 (50%), and 5 (7.6%) in age groups <1 year, 1–10 years, and 11–30 years old, respectively (Table 1). No influenza viruses were detected from patients in age group 31–50 years old. Out of the 28 patients from the age group <1 year, 19, 8, and 1 patients were infected with type A, B and C, respectively. From this age group, the results of detecting other respiratory viruses from patients negative for influenza viruses, showed that 17 (81%) and 4 (19%) patients were positive for RSV, and rhinovirus, respectively (Table 3). In age group 1 – 10 years old, a total of 204 samples (Table 1) were screened and of the 33 patients who proved positive for influenza, 13, 17, 1, and 2 had type A, type B, type C and a co-infection of types A and B respectively. From patients negative for influenza virus and belonging to the age group 1 – 10 years, a total of 20 patients were found positive for other respiratory viruses: nine were positive for RSV , nine for rhinovirus and two for adenovirus (Table 3).

From patients of the age group 11 – 30 years, three samples were positive for influenza type A and two for influenza type B (Table 1). From patients of this same age group, negative samples for influenza virus were tested for other respiratory viruses and one patient was found positive for RSV and one for rhinovirus (Table 3). From the age group 31 to 50, no sample was found positive for influenza and other respiratory viruses (Tables 1 and 3).

According to the gender, influenza viruses were detected in 39 (59%) male and 27 (41%) female patients. Other respiratory viruses were detected in 23 (53.5%) male and in 20(46.5%) female patients (Table 4).

**Table 4:** Patients with acute respiratory infection, classified as to gender and the respiratory virus

**Virus detected**	** Number (%)**		
	**Male**	**Female**	**Total**
Influenza viruses			
Influenza A	19	16	35
Influenza B	16	11	27
Influenza C	2	0	2
Influenza A and B	2	0	2
Total	39 (59.0)	27 (41.0)	66 (100)
Other respiratory viruses			
RSV	13	14	27
hMPV	0	0	0
Rhinovirus	8	6	14
Adenovirus	2	0	2
Total	23 (53.5)	20 (46.5)	43 (100)

### Nucleotide sequence and phylogenetic analysis of influenza viruses

**Figure 1:**
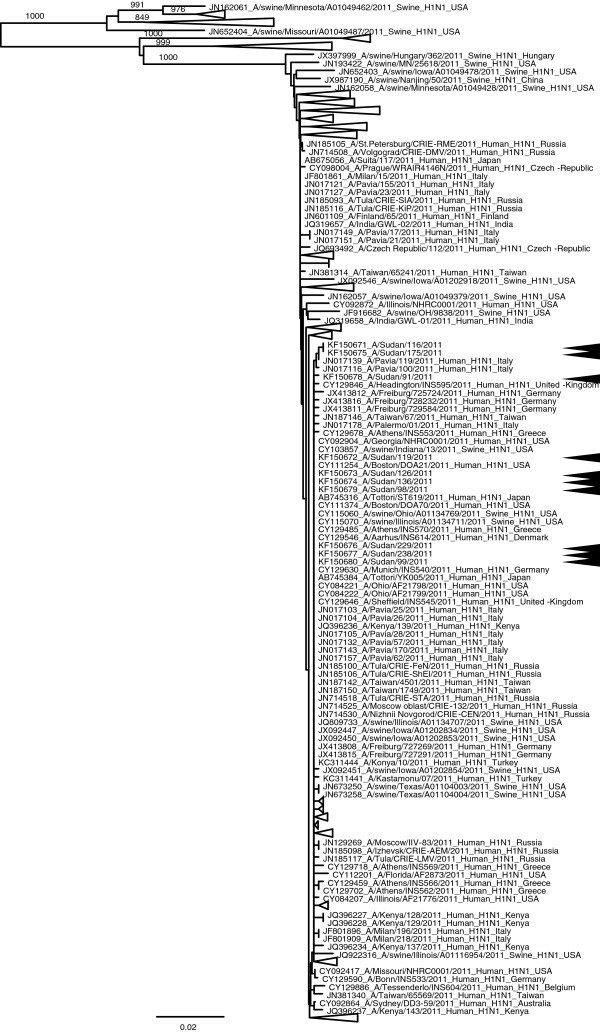
**Phylogenetic tree of HA1 gene of influenza A strains from Sudan.** Tree was constructed from representative strains detected in 2011. Some branches were collapsed for clarity. The actual tree with all the strains can be viewed in Additional file 4 and the strains in the expanded branches are indicated by green lines. Bootstrap values (1000 times) are shown at the nodes. Arrowheads indicate strains from Sudan.

**Figure 2:**
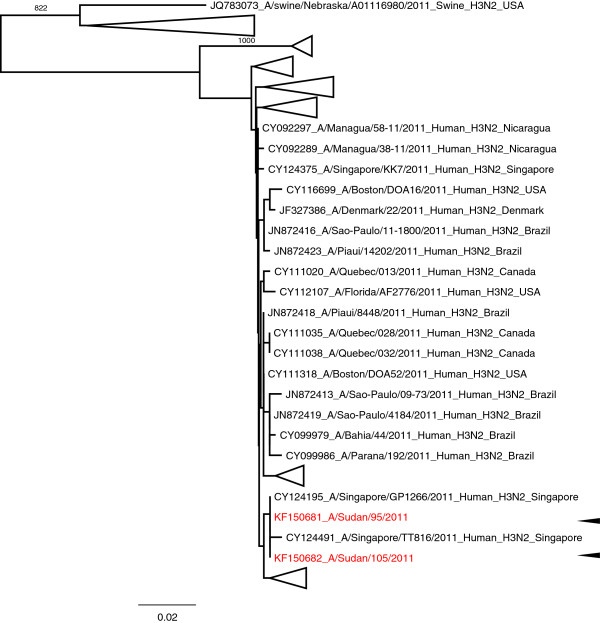
**Phylogenetic tree of HA3 gene of influenza A strains from Sudan.** Tree was constructed from representative strains detected in 2011. Some branches were collapsed for clarity. The actual tree with all the strains can be viewed in Additional file 5 and the strains in the expanded branches are indicated by green lines. Bootstrap values (1000 times) are shown at the nodes. Arrowheads indicate strains from Sudan.

**Figure 3:**
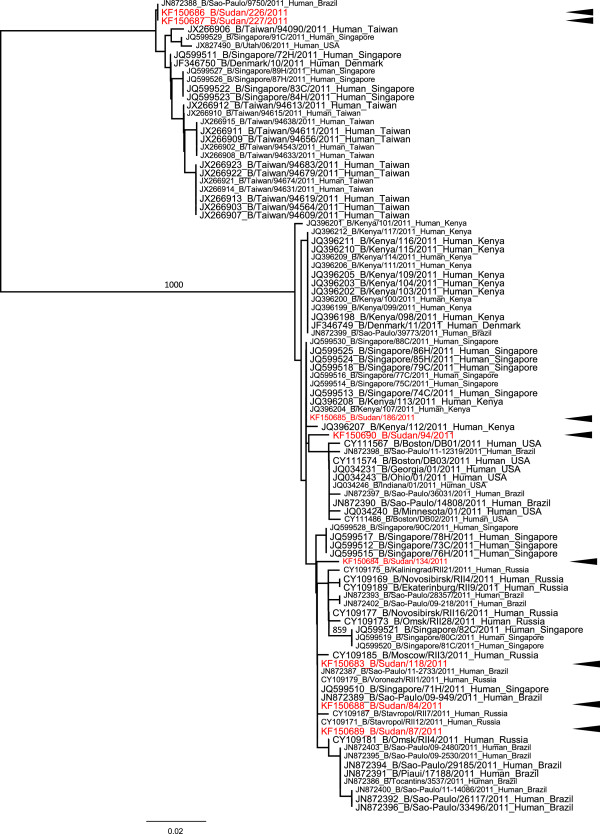
**Phylogenetic tree of HA gene of influenza B strains from Sudan.** Tree was constructed from representative strains detected in 2011. Bootstrap values (1000 times) are shown at the nodes. Arrowheads indicate strains from Sudan.

Successful sequence of H1 of A(H1N1)pdm09 subtype, influenza type A H3 and influenza type B based on HA gene was done on 20 samples—10 samples of A(H1N1)pdm09, 2 samples of H3 and 8 samples of influenza type B—with the RNA template either directly from throat swab samples or from virus isolates (8 isolates) present in infected culture fluid (Table 5). The nucleotide sequences were compared with other sequences published in GenBank. Phylogenetic trees that were generated for the H1 of A (H1N1) pdm09, influenza types A H3 and influenza virus type B are shown in Figures 1, 2 and 3, respectively, and the aligned sequences of Sudan strains in fasta format are found in the additional materials (Additional files 1, 2 and 3). The phylogenetic tree on H1 (Figure 1; Additional file 4 for actual phylogenetic tree) shows that they belong to the same clade and are related to several strains from around the world (USA, Japan, Italy, United Kingdom, Germany, Russia, Greece, Denmark, Taiwan, Turkey and Kenya). Seasonal H3 subtypes detected only during the 2011 season have close similarity to strains from several parts of the world (Singapore, Brazil, Canada, Denmark, USA and Nicaragua; Figure 2, Additional file 5) also. The phylogenetic tree for the influenza B (Figure 3) shows two different populations of this virus in Sudan strains detected in 2011 and these populations are related to the strains in Kenya, USA, Brazil, Russia, Taiwan and Singapore.

**Table 5:** Influenza viruses that were sequenced and related information

**Strain name**	**Date of collection**	**Age**	**Sex**	**GenBank accession number**
A/Sudan/99/2011 (H1)	2011-01-20	2 mo	M	KF150680
A/Sudan/98/2011 (H1)	2011-01-20	8 mo-4 d	M	KF150679
A/Sudan/136/2011 (H1)	2011-01-30	6 mo	F	KF150674
A/Sudan/126/2011 (H1)	2011-01-26	9 mo	M	KF150673
A/Sudan/119/2011 (H1)	2011-01-22	3 mo	M	KF150672
A/Sudan/91/2011 (H1)	2011-01-21	8 mo	F	KF150678
A/Sudan/116/2011 (H1)	2011-01-22	6 yr	F	KF150671
A/Sudan/175/2011 (H1)	2011-02-01	2 mo	F	KF150675
A/Sudan/238/2011 (H1)	2011-02-14	4 yr-5 mo	F	KF150677
A/Sudan/229/2011 (H1)	2011-02-13	4 yr-8 mo	F	KF150676
A/Sudan/95/2011* (H3)	2011-01-20	5 yr	M	KF150681
A/Sudan/105/2011 (H3)	2011-01-22	2 mo	M	KF150682
B/Sudan/134/2011	2011-01-30	10 yr	M	KF150684
B/Sudan/118/2011	2011-01-22	7 d	M	KF150683
B/Sudan/84/2011	2011-01-19	11 yr	M	KF150688
B/Sudan/87/2011	2011-01-19	4 mo	F	KF150689
B/Sudan/94/2011	2011-01-20	5 yr	M	KF150690
B/Sudan/186/2011	2011-02-06	4 mo	M	KF150685
B/Sudan/226/2011	2011-02-13	1 yr-1 mo	F	KF150686
B/Sudan/227/2011	2011-02-13	5 mo	M	KF150687

*Source of the virus also contained influenza B.

## Discussion and conclusion

Little is known about the epidemiology of influenza viruses in Sudan in particular and in Africa in general. As a consequence, authorities in countries located just on either side of the equator in the sub-Sharan Africa find it difficult to decide which influenza vaccine to choose: the northern hemisphere or the southern hemisphere [7,8]. In Sudan, no regular program of vaccination against seasonal influenza is being implemented [9]. For the 2013–2014 northern hemisphere influenza season [7] and the 2013 southern hemisphere influenza season [8], World Health Organization recommended that the composition of trivalent vaccines include an A/California/7/2009 (H1N1) pdm09-like virus and in addition other viral components that are specific only for each type e.g. a B/Massachusetts/2/2012-like virus for the northern hemisphere vaccine and B/Wisconsin/1/2010-like virus for southern hemisphere vaccine. Hence, there is a need to know the type of influenza viruses occurring in a particular geographical area, especially in those areas with uncertain boundaries, to serve as a guide in choosing a specific type of vaccine. Through surveillance in sentinel hospitals, data can be provided for describing influenza epidemiology and seasonality, for characterizing circulating strains of influenza virus to serves as a basis in selecting the right type of vaccine and for monitoring influenza pandemics [10].

Our current study in Sudan is one of the first to report directly-measured rates of influenza-associated hospitalization in adults and children. The study revealed that influenza types A, B and C were circulating in Sudan, and were detectable in 16.5% of the patients. Types A and B were responsible for most of the influenza infections. The subtype influenza A(H1N1)pdm09 was detected in 75.7% of the patients with influenza A infection. Type C was detected in patients only during the 2011 season. Influenza virus infections were detected in all age group examined but with the exception of the oldest group (30–50 years). Infection was recorded mostly in the young age groups (<1 year, 1–10 years). Taken all of the above findings into consideration, these may indicate complex epidemiology of influenza viruses in Sudan. Similar results were recorded in Nigeria, where all 3 types of influenza viruses were also detected [11]. A study conducted in Sudan during two periods (December 1987 to April 1988 and September 1990 to March 1991) reported the detection of influenza A (or B) in four out of 213 (2%) children by using either immunofluorescence assay for antigen detection or ELISA [12].

Phylogenetic analysis of H1 of A(H1N1)pdm09 subtypes in this study indicated that they belong to the same clade and that they were related to the strains in the USA, Japan, Italy and other several countries. Seasonal influenza A H3 subtypes were similar to strains from Singapore as well as from other parts of the world. Influenza B strains from Sudan belong to two different world-wide distributed clades. This pattern suggests that Sudan strains were a part of worldwide endemic population.

Other respiratory viruses including RSV, rhinovirus and adenovirus were also found to cause respiratory infection mostly (95.4%) in the younger age groups (<1 year, 1–10 years). Infection by RSV and adenovirus among Sudanese children was also reported previously [12]. Interestingly, no hMPV was detected in the present study; this virus has been detected in other countries in Africa including Kenya [13] and Nigeria [11]. These findings highlight the need for the establishment in Sudan of rapid, sensitive, and specific diagnostic techniques (such as ones used here) for better management of respiratory infections especially in the high risk groups (infants, elderly and immunocompromised patients).

To our knowledge this is the first attempt to identify the causative viral agents of respiratory infections in Sudan by using molecular techniques and to characterize influenza viruses specifically. Finally, the results obtained should call for wider surveillance at the national level in order to fully elucidate the true status and epidemiology of influenza viruses in Sudan.

## Methods

### Study site

The study was conducted in the Khartoum State of Central Sudan, which had a population of 5,274,321 at the time of the 2008 national census [14]. Patients, including hospitalized patients and children seen at the emergency department in Khartoum Hospital, were recruited between January to March in 2010 and in the same months in 2011.

### Data collection

Through a structured questionnaire, information on age, occupation, gender, onset of disease, clinical symptoms, response to antibiotics, influenza vaccination and place of sample collection, was recorded for each patient during the medical consultation.

### Collection of specimens

A throat swab sample was obtained from each patient by inserting sterile nylon swab (Regular Flocked swab, Cat. No. 520CS01, Copan Diagnostics Inc., Murrieta, Calif, USA) and rubbing the tonsils and the posterior wall of the pharynx. Samples collected were transported in an ice pack on the same day of collection to the laboratory of the Department of Virology, Central Laboratory (Ministry of Science and Technology) and stored at −80°C until tested.

The frozen aliquots were transported on dry ice to the Department of Virology, Institute of Tropical Medicine, Nagasaki University, Japan, where one aliquot per enrolled patient was tested for the presence of influenza virus by means of multiplex-real time RT-PCR.

### RNA extraction

Total RNA was extracted by using the QIAamp Viral RNA Mini spin according to the protocol of the manufacturer (Qiagen, Germany). Briefly, 140 μl of throat swab sample was added to 560 μl buffer AVL containing carrier RNA, and then incubated at room temperature for 10 minutes. Subsequently, 560 μl of ethanol (96-100%) was added to the sample after which 630 μl of the resulting solution was applied to a column. A volume of 500 μl of AW1 and AW2 was added for washing and the nucleic acids were eluted with 60 μl AVE buffer and stored at -80°C until used.

### Real-time RT-PCR

Real-time one step RT-PCR was done to detect viral RNA by using a commercial kit following the manufacturer’s instructions (One-Step Real-Time RT-PCR Master Mixes Kit, Invitrogen, USA). Multiplex real-time RT-PCR was carried out by using 3 primer/probe sets as shown in Table 6 (set 1: Inf A [15]; Inf B [16]; Flu C [this study] and RNase P [15]; set 2: RSV [16], hMPV [17], Rhino [18] and Adeno [16]; set 3: SW Inf A [15] and HA3 [19]. With the primer/probe set 1, RNaseP was used as an internal control and real-time PCR was carried out following the protocol (but with modification for multiplex procedure) provided by Centers for Disease Control and Prevention, USA (CDC) [15]. The real-time PCR master mix for one reaction was prepared as follows: 4 μl of 5X PCR reaction mix (consisting of a proprietary buffer system, MgSO_4_, dNTPs ,and stabilizers), 6 μl of primer/probe, 1 μl of 1U enzyme mix, 5.5 μl of molecular grade water, 0.5 μl of 10X ROX and 3 μl of total RNA (90.3 ng/μl). The final volume was 20 μl for a single reaction. The reaction was performed in an automated 7500 real-time PCR (AB Applied Biosystems, USA). The thermal cycling conditions were 15 minutes at 50°C for reverse transcription, 2 minutes at 95°C for initial denaturation and 45 cycles of 15 seconds at 95°C for denaturation and 45 seconds at 60°C for annealing and extension. A sample whose growth curve crossed the threshold line within 40 cycles (Ct < 40) was considered as positive. For identifying influenza A (H1N1) pdm09, positive results must be obtained with InfA in set 1 and SW Inf A in set 3 primer/probe.

### Virus isolation

Throat swabs samples found positive for influenza viruses by real time RT-PCR underwent viral culture. Samples were first clarified by using syringe filter (0.22 μm). A 500 μl volume of each sample was then inoculated into corresponding 25 cm^2^ cell culture flask containing confluent Madin Derby canine kidney cells (MDCK), provided by the Department of Virology, Institute of Tropical Medicine, Nagasaki University, Japan. These inoculated cells were maintained in minimal essential medium (MEM) without fetal bovine serum but with trypsin (0.25 mg/ml), incubated at 37°C for 7 days, and examined daily for cytopathic effect (CPE). Multiplex RT- real-time PCR for influenza viruses types A, B and C was then carried out as described above on RNA extracted from the infected culture fluid to confirm CPE.

### Sequence and phylogenetic analysis

Reverse transcription was performed on extracted RNA by using Superscript III Reverse Transcriptase (Invitrogen) and random hexamers, following RNA denaturation at a temperature of 95°C over a period of 5 min. PCR was conducted by using TaKaRa LA Taq DNA polymerase (TaKaRa Bio, Inc. Otsu, Japan) with primers targeting the HA gene of influenza A virus H1 [20], influenza A virus H3 [20] and influenza B virus [21], respectively (Table 6). In influenza A virus H1 type, the 699 bp corresponding to 461–1159 nts in the segment 4 of strain A/Puerto Rico/8/34(H1N1) (GenBank:NC_002017) region was used to build the phylogenetic tree. In influenza A virus H3 type and in influenza B virus, the 480 bp corresponding to 115–594 nts in the segment 4 of A/New York/392/2004(H3N2) (GenBank:NC_007366), and the 260 bp corresponding to 1332–1591 nts in the segment 4 of strain B/Lee/40 (GenBank:NC_002207) were used, respectively. The sequences were aligned by MAFFT version 7.023b [22] with other influenza strains isolated in 2011, obtained from the influenza virus resource at the National Center for Biotechnology Information [23]. Phylogenetic trees were constructed by neighbor-joining method [24] using clustal × 1.83 [25] Trees were drawn by FigTree version 1.4.0 [26]. For influenza virus types A H1, AH3, and B, the number of strains used to build the phylogenetic trees was 856, 603, and 96 respectively.

**Table 6:** Primers and probes used in this study*

**Primer Name**	**Sequence (5′ to 3′)**	**Target Gene**	**Reference**
	**Primers for real time RT-PCR**		
**Set 1**			
Inf A- F	GACCRATCCTGTCACCTCTGAC	M	[15]
Inf A-R	AGGGCATTYTGGACAAAKCGTCTA		
Inf A- probe	TAMRA-TGCAGTCCTCGCTCACTGGGCACG-BHQ2		
InfB-F	ATCGGATCCTCAACTCACTCTT	NS	[16]
InfB-R	TGACCAAATTGGGATAAGACTC		
InfB-probe	FAM-CTCGAATTGGCTTTGRATGTCCTTCAT-BHQ1		
FluC-F	TGCATTAAAAGCGGATTCGTT	NS	this study
FluC-R	GCTGGGCTCCTGATTTGATACT		
FluC-probe	HEX-TGGCTACCGATGAAATCTCTCTCACTATACT-BHQ1		
RNase P-F	AGATTTGGACCTGCGAGCG	RNase P	[15]
RNaseP-R	GAGCGGCTGTCTCCACAAGT		
RNaseP-probe	CY5-TTCTGACCTGAAGGCTCTGCGCG-BHQ3		
**Set 2**			
RSV-F	GCCAAAAAATTGTTTCCACAATA	L	[16]
RSV-R	TCTTCATCACCATACTTTTCTGTTA		
RSV- probe	FAM-TCAGTAGTAGACCATGTGAATTCCCTGCA-BHQ1		
hMPV-F	AACCGTGTACTAAGTGATGCACTC	N	[17]
hMPV-R	CATTGTTTGACCGGCCCCATAA		
hMPV-Probe	Cal Flour Orange560-CTTTGCCATACTCAATGAA CAAA CT-BHQ1		
Rhino-F	TGGACAGGGTGTGAAGAGC	5′- UTR	[18]
Rhino-R	CAAAGTAGTCGGTCCCATCC		
Rhino-Probe	TAMRA-TCCTCCGGCCCCTGAATG-BHQ2		
Adeno-F	GCCCCAGTGGTCTTACATGCACATC	H	[16]
Adeno-R	GCCACGGTGGGGTTTCTAAACTT		
Adeno-Probe	Quasar670-TCGGAGTACCTGAGCCCGGGTCTGGTGCA-BHQ2		
**Set 3**			
SW InfA -F	GCACGGTCAGCACTTATYCTRAG	Np	[15]
SW InfA-R	GTGRGCTGGGTTTTCATTTGGTC		
SW InfA-Probe	HEX-CYACTGCAAGCCCA“T”(BHQ1)ACACACAAGC AGGCA		
HA3-115-F	GCTACTGAGCTGGTTCAGAGTTC	H3	[19]
HA3-375-R	GAAGTCTTCATTGATAAACTCCAG		
HA3-208-Probe	FAM-CTATTGGGAGACCCTCATTGTGATGG-BHQ1		
	**Primers for sequence**		
H1-F	AGCAAAAGCAGGGGAAAATAA	H1	[20]
H1-R	GCTATTTCTGGGGTGAATCT		
H3-F	AGCAAAAGCAGGGGATAATTC	H3	[20]
H3-R	TGCCTGAAACCGTACCAACC		
Inf B-F	AGCAGAAGCGTTGCATTTTC	H3	[21]
Inf B-R	ACCAGCAATAGCTCCGAAGA		

*Abbreviations: *M* Matrix, *NS* Nonstructural, *RNase P* Ribonuclease P, *L *L protein, *N* nucleocapsid protein, *Np* nucleoprotein, *UTR* untranslated region, *H* hexon protein, *H1* haemagglutinin subtype 1, *H3* haemagglutinin subtype 3.

### Ethical review

The study was approved by the Ethical Review Committee (ERC) of the Ministry of Health Khartoum State, Sudan. Informed consents were obtained from adult patients, or from parents or legal guardians of children.

## Competing interests

Authors have no competing interests to declare.

## Authors’ contributions

KAE did the sample collection, virus isolation, multiplex real-time RT-PCR, and drafted the manuscript. TN designed the experiments for sequence and did the phylogenetic analysis. TK designed the primers for detecting influenza C virus, gave some suggestions for virus isolation and supervised the work in multiplex real-time RT-PCR. CCB helped in the analysis of data and worked in the preparation and edition of the manuscript. ARMEH, IME and EAGK contributed to the conception and design of the study and helped in the drafting of the manuscript. KM contributed to the conception and design of the study, the drafting of the manuscript and the work on virus isolation. All authors read and approved the final manuscript.

## Supplementary Material

Additional file 1 Fasta format of nucleotide sequence of HA1 gene of different strains of influenza A virus (found in GenBank and reported in this study). Click here for file

Additional file 2 Fasta format of nucleotide sequence of HA3 gene of different strains of influenza A virus (found in GenBank and reported in this study). Click here for file

Additional file 3 Fasta format of nucleotide sequence of HA gene of different strains of influenza B (found in GenBank and reported in this study). Click here for file

Additional file 4 Actual phylogenetic tree of HA1 gene showing all influenza A virus strains. Click here for file

Additional file 5 Actual phylogenetic tree of HA3 gene showing all influenza A virus strains. Click here for file
