# Supplementary figures and images for: Survey of causative agents for acute respiratory infections among patients in Khartoum- State, Sudan, 2010–2011
Source: Virol J. 2013 Oct 25;10:312. doi: 10.1186/1743-422X-10-312 (PMC3831848; doi:10.1186/1743-422X-10-312)

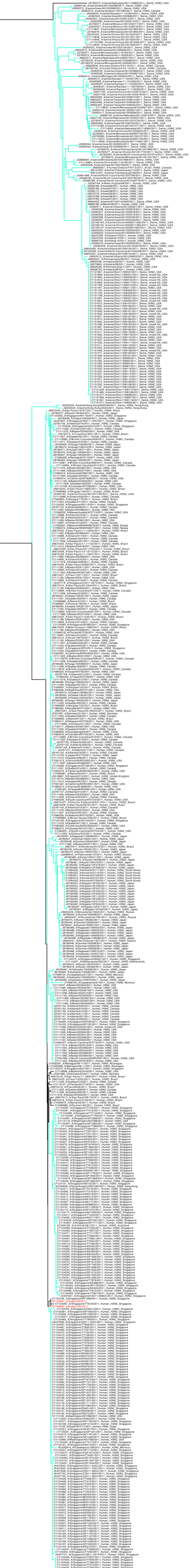

Supplement: Additional file 5 — Actual phylogenetic tree of HA3 gene showing all influenza A virus strains. [file 1743-422X-10-312-S5.pdf]
